# ‘Containment, delay, mitigation’: waiting and care in the time of a pandemic

**DOI:** 10.12688/wellcomeopenres.15970.2

**Published:** 2020-09-10

**Authors:** Lisa Baraitser, Laura Salisbury

**Affiliations:** 1 Department of Psychosocial Studies, Birkbeck University of London, London, WC1E 7HX, UK; 2 Department of English and Film, University of Exeter, Exeter, EX4 4QJ, UK

**Keywords:** COVID-19, UK Government, waiting, time, care, violence, psychoanalysis, World War II

## Abstract

In this paper, we take up three terms – containment, delay, mitigation – that have been used by the UK Government to describe their phased response to the COVID-19 pandemic. Although the terms refer to a political and public health strategy – contain the virus, flatten the peak of the epidemic, mitigate its effects – we offer a psychosocial reading that draws attention to the relation between time and care embedded in each term. We do so to call for the development of a form of care-ful attention under conditions that tend to prompt action rather than reflection, closing down time for thinking. Using Adriana Cavarero’s notion of ‘horrorism’, in which violence is enacted at precisely the point that care is most needed, we discuss the ever-present possibility of failures *within* acts of care. We argue that dwelling in the temporality of delay can be understood as an act of care if delaying allows us to pay care-ful attention to violence. We then circle back to a point in twentieth-century history – World War II – that was also concerned with an existential threat requiring a response from a whole population. Our purpose is not to invoke a fantasised narrative of ‘Blitz spirit’, but to suggest that the British psychoanalytic tradition born of that moment offers resources for understanding how to keep thinking while ‘under fire’ through containing unbearable anxiety and the capacity for violence in the intersubjective space and time between people. In conditions of lockdown and what will be a long and drawn-out ‘after life’ of COVID-19, this commitment to thinking in and with delay and containment might help to inhabit this time of waiting – waiting that is the management and mitigation of a future threat, but also a time of care in and for the present.

## Introduction

On 12 ^th^ March 2020, Professor Chris Whitty, the UK Government’s Chief Medical Advisor, stated in a news conference: ‘We are entering a delay phase’. Global ‘containment’ of novel coronavirus, first detected in Wuhan, China, had not worked and this was now a crisis. COVID-19 was spreading, with Europe as its new epicentre. The UK’s own ‘containment’ phase of its domestic strategy – testing, quarantine and the tracing of known contacts with a patient – was soon abandoned and the UK abruptly moved into the more obviously temporal ‘delay’ phase of the public health operation ( Policy Paper, 2020). Processes of social distancing, self-isolation and then, ultimately, ‘lockdown’ were instigated in an attempt to lengthen and flatten the peak of the outbreak and reduce the number of cases *at any one time*. This, it was hoped, would give the health service a chance of survival and help to manage the outbreak in a population assumed to be unable to cope with more than 12 weeks in isolation. ‘Timing’, as Whitty put it, was ‘everything’ ( Whitty, 12 March, 2020). Yet, as one National Health Service (NHS) consultant put it as early as 16 ^th^ March, despite a month of planning ‘what has blindsided us is the speed at which the hypothetical became real and then became obsolete’ ( Anonymous, 2020a). The increase in cases happened so rapidly in a system already operating at almost total capacity after a decade of austerity that, according to this anonymous report, by 16 ^th^ March the system was already overwhelmed, even though the UK Government claimed in mid-April that hospitals were still running ‘below their ceiling’ ( Whitty, 13 April, 2020). But with cancelled operations and outpatient appointments now pushed not into a planned future but a suspended time that cannot easily be held in mind, it will take time to know about the full secondary health effects of COVID-19 and the results of the Government’s interventions. There will be a cascade of impacts on the economy and the NHS that will affect the delivery of timely healthcare for years to come.

Everywhere we look, the commentary on the COVID-19 pandemic focuses on the question of time and timing. These questions include: how to make timely interventions – acting swiftly and decisively while also trying to instigate practices of waiting and delaying; when to instigate and when to end lockdowns that suspend and transform the temporalities of work, sociality and economic and political activity that play out in acutely uneven ways; how to implement systems that wait for ‘the hypothetical’ and then are flattened almost immediately; ^1^ managing phenomenological experiences in isolation that give rise to time cycling or becoming sluggish or of being ‘outside of time’; and the prospect of the deep violence of the effects of governmental responses to the virus that will not been known about for decades. Although the strategy of ‘containment, delay and mitigation’ suggests a linear temporality that seems to echo something like the progression of a disease, the experience of living with and through these phases has suggested a much less straightforward set of temporal experiences. Just as diseases themselves frequently have much more complex trajectories that include suspensions, remissions, recursions, set-backs and recurrences, it has been hard to know precisely which phase of the strategy we might be inhabiting at any moment, or whether it is either practical or ethical to imagine one term superseding the last.

As humanities and social science scholars working on histories and experiences of waiting in and for healthcare, we are concerned to understand how questions of time intersect with those of care in these current times. What are the discourses of care being ostensibly offered by ‘containment, delay and mitigation’? The mantra that has emerged in the UK has been ‘stay at home; protect the NHS; save lives’. The explicitly temporal strategy of delay, from where we are currently writing, indeed invokes a call for care for an institution that on the one hand retains a particular place in the British cultural imaginary (‘our’ NHS, as Boris Johnson now repeatedly names it) ^2^, yet on the other is routinely described, and experienced by those working within it, as ‘dying’. Particularly since the reforms of 2013, the NHS has persistently been represented as staggering on in an ongoing and enduring crisis brought on by chronic underfunding, creeping privatisation and a withdrawal from Europe that has already led to further staff shortages, demoralisation and burnout of staff at every level.

Public debate has aligned some aspects of the Government’s strategy, particularly in its initial articulation, with dangerous inaction, while the Government has insisted that the ‘delay’ we are now in is a form of care, especially for the most vulnerable. We would like to articulate an alternative view in which delay holds within itself the possibility for care, but only insofar as it must also ‘know’ about violence: violence that might express itself in knowingly ‘letting’ certain groups of people die; in exposing vulnerability to shame rather than support; or in denying responsibility for political decisions that have kept the NHS running in permanent crisis. The violence we mean to talk about, in other words, is not just enacted through social structures or systems, but also through systemically denying care to those who need it. These are forms of social violence that entail the intentional use of power that results in harm through failures of care, although they are not always recognised in these terms. We argue, here, that knowing about these forms of violence relies on using the temporality of delay to pay care-ful attention over time to the possibility of harm in states of extreme vulnerability and powerlessness. To do this we must move in the counter-direction to the UK’s strategy (containment to delay to mitigation) and instead begin in delay. From there we will work ‘backwards’ to understand ‘containment’ through a psychoanalytic lens, in order to finally offer some thoughts on what mitigation of harm might mean in a (post) COVID-19 context.

## Delay

In the opening phase of the UK Government’s strategy of ‘delay’, the notion of building ‘herd immunity’ emerged under the auspices of a care for ‘lives’ and protection for the ‘most vulnerable’ – those over the age of 70 and those with ‘underlying health conditions’. But there was already a tense relation here between different temporalities. As Boris Johnson suggested in a much-circulated interview on 5 ^th^ March: ‘One of the theories is that perhaps you could take it on the chin, take it all in one go and allow the disease, as it were, to move through the population, without taking as many draconian measures’ ( *This Morning*, 5 March 2020). In other words, delay might require some populations, seemingly those less likely to suffer the most severe effects of the virus, to be exposed *without* delay, while the most vulnerable were shielded – contained within their homes. Targeted containment and delay, which was never fully actualised as a policy, was linked to an idea of ‘strik[ing] a balance’ ( *This Morning*, 5 March, 2020) between relatively minor interventions, such as advice on hand-washing and moderate social distancing, and the more ‘draconian’ strategy of lockdown ^3^. Yet, as was quickly established, the political discourse that took up the epidemiological modelling underpinning this strategy dangerously condoned a form of thinking in which some lives – the elderly, the chronically ill and the disabled – were deemed more expendable than others. For many, this particular configuration of ‘delay’ was experienced as a form of inaction that seemed all too clearly underwritten by an ongoing form of social violence familiar to populations whose lives have been framed as not of equal value and somehow ‘ungrievable’, to use Judith Butler’s formulation ( Butler, 2004; Butler, 2019). As the Black feminist poet Audre Lorde has stated: ‘some of us were never meant to survive’ ( Lorde, 1978, p. 31).

Can delay then be felt as care; is it indeed care, or is it a form of abandonment as some are arguing ^4^ – an abandonment of those most in need of care? As is now emerging, those who need care include those who contract the virus; the healthcare workers who care for them but who may themselves require care; those affected by the severe and lasting effects of an economy under lockdown; those who find themselves trapped at home in situations that are physically and mentally dangerous; those already living in food poverty or without homes and unable to self-isolate; those in care homes; those in prison; those in forms of work deemed essential despite the lack of provision for safe working; or those forced to make impossible choices between work and acute states of poverty. If it is a form of abandonment at the point that care is most needed, then it constitutes what the philosopher Adriana Cavarero has called ‘horrorism’ ( Cavarero, 2009). Horrorism is Cavarero’s term for a form of violence that offends the human subject at an ontological rather than socio-political level. It describes a form of violation of another that occurs when that other opens themselves, or finds themselves open, or is compelled to make themselves open to both care and harm at the same time. An infant might be a paradigmatic figuration of this form of vulnerability, one in which dependency on care for survival, for going on being, necessarily opens the infant to the potential of harm that would do ontological violence if it were enacted, but in a (post) COVID-19 world, so too are many others: keyworkers with no protective equipment; detainees who already face shortened life expectancies; children who depend on school to provide the only meal of the day; and, as is increasingly becoming clear in the global north, people of colour – whether those working life-long in the UK heath service who represent almost half of all medical professionals, or those in the US living in urban centres and who, due to enduring conditions of racism, have a higher likelihood of not being able to access to healthcare. Care, in these cases surely must avoid horrorism. It must not, however unwittingly, inflict harm at the very point that care is needed.

We can think of care broadly as a set of social capacities: those that are necessary for birthing and raising children; for sustaining and maintaining kinship groups and community connections; and forms of social reproduction that underpin every aspect of capitalism’s proliferation that have always been gendered, classed and racialized – women’s work, poor women’s work, poor women of colour’s work ( Baraitser, 2017). Although we can and should pay close attention to ‘state care’ or ‘caring economies’ ^5^, the often mundane temporalities of socially reproductive labour – temporalities of waiting, repeating, staying, returning, maintaining, enduring, persisting – that involve *not* moving on are easily overlooked. Indeed, they are sometimes set against the heroic exactitude of the timeliness of healthcare: care in acute situations such as cardiac arrest, surgery and A&E settings, even though the majority of day-to-day healthcare practices have elongated temporalities at their core. Consider the ‘watchful waiting’ routinely used in general practice in which a patient and practitioner must wait to see if and how a symptom develops or whether a medication takes hold; the slow unfolding of trust required to communicate psychological distress that forms a vital part of the therapeutic alliance in mental health treatment; or the uncertain and unknowable time of palliative care at the end of life. Even Boris Johnson, not always known for his attention to detail, was able to acknowledge that during the 48 hours in intensive care at St. Thomas’ hospital, London, it was the minute-by-minute watchful waiting of two nursing staff, Jenny McGee and Luís Pitarma, that enabled his recovery and, in his terms, ‘saved my life’ ( Johnson, 2020b). When we overlook care that takes time, or is itself a practice that waits to see what giving time to a situation may bring, we enact the antithesis of care. We fail to think carefully about care.

What we might say, then, about care is twofold: that it is bound up in particular ways with enduring time, and that it requires a form of knowing-about, or thinking-about, the antithesis of care – failures to care, horrorism or the perverse pull to enact harm when care is most needed. We want to argue that these failures can, if we can pay attention to them, bring on new ways of thinking – forms of ‘care-ful attention’ whose temporal forms are waiting, staying, maintaining, enduring, returning, repeating and persisting. Care, from this perspective, is not just a relational practice that develops over time, or one that takes time; it is a practice that *produces* time in conditions that are otherwise felt to be stuck and unable to change. In her discussion of what she calls ‘care time’, Maria Puig de la Bellacasa elaborates how care both takes time and involves ‘making time of an unexceptional particular kind’ ( Puig de la Bellacasa, 2017, p. 206). Although, affectively, care time can be enjoyable, she writes, it is also ‘very tiresome, involving a lot of hovering and adjusting to the temporal exigencies of the cared for’ (p. 206). Much care in an intensive care unit takes just this form. Care time, as Puig de la Bellacasa states, is not future-orientated or a matter of righting past wrongs, but ‘suspends the future and distends the present’ (p. 207). It produces the time for care-ful attention by pushing back on the anticipated joys or indeed horrors of the future, the pleasures of the present or the accumulated regrets of the past. In this sense *care time is the time of delay*.

The meaning of delay in English hovers between two contradictory impulses: to put off or defer action, so that delay opens up the time of lingering, loitering, dithering or procrastinating; and a more forceful impulse that has to do with detaining, holding up, making late and hindering progress ( *OED*). On the one hand, delaying puts aside the future in the name of a temporal hiatus that slows the time of progress and appears to offer an approach to present time that might make it possible to grasp it; on the other, delay remains futural – the possibility of deferral is precisely premised on the yet-to-come, on what Jacques Derrida calls the ‘ *a-venir’*. For instance, for Derrida, the relational encounters of hospitality, justice and mourning all retain their ethical potential through the necessity of their postponement, their delay ( Derrida, 1992; Derrida, 1994; Derrida, 1995). And in the realm of politics, for Derrida, there is an imperative for democracy to function through this delayed temporality in order that it remains open to revision and resists the closure of identity in which all difference is eradicated ( Derrida, 1997). In the French etymology, there is an even clearer distinction between waiting as an interval that intervenes in the flow of time ( *dans un délai*) and an excessive slowness or being behind the times ( *retard*, *attardé*). We could say that if there is an agony in delay that is distinct from simply waiting, it is this awareness that despite the desire to foreclose the future and push back the past, to loiter and linger and dwell in the delay, there remains a temporal drag that nevertheless insists on a relation between past and future. The present is never ‘free time’, in other words – freed from its obligations to a future based on its experience that it is always already past. Delay, rather, reveals how the present drags with it a past that is always already obliged to a future. In this sense care that entails hovering and adjusting is already weighed down with its cultural and historical situatedness, its past lives that it cannot shake off.

What might it mean to go on knowing about the kinds of vulnerability to social violence precipitated by COVID-19 in the temporality of delay? In *Delay of the Heart*, the final part of David Appelbaum’s three-volume philosophical meditation on time and ethics ( Appelbaum, 2001), he elaborates delay as closely bound up with knowing and the problems of the closure of thought – with the way that thought both remembers and projects into a future, but is unable to inhabit the present. For Appelbaum, as for Locke, thought is essentially retentive in its ‘grasping again what was once present’ ( Appelbaum, 2001, p. 2), in retrieving conditions from the past and projecting them into the future. From this perspective, cognition is parthenogenetic, in the sense that it gives birth only to more of itself, more thought. This is thought’s primary concern: to reproduce the conditions of its own reproduction through the smooth and uninterrupted operation between retention and projection.

But, for Appelbaum, such thought misses something fundamental that becomes visible in the temporal hiatus we call delay. In delay it appears initially that there are two positions of experience. From the perspective of the one lagging behind there is no delay, there is only the other who has pulled away at a pace that produces a discrepancy and who cannot now inhabit the place of being ‘behind’. We can be delayed, but it is the other who waits for us. Delay from this position is denied. From the perspective of the one ahead, delay is a fact: there is another who lags. Delay therefore produces two modes of thought: denial and fact. But for Appelbaum, there is a third position of experience that entails neither fact nor denial. He calls this the ‘view from the heart’ which breaks into the smooth running and endless flow of thought. Delay of the heart is the introduction of a somatic element, the heart, into the sphere of cognition. It arrests thought and allows a different form of judgment to emerge, allowing delay to ‘weigh’ a situation differently from the procedures and logics of thought ( Applebaum, 2001, p. 5). Appelbaum reminds us that the root meaning of delay is *laxare*, to relax or decontract (p. 7). In delay, something in thought slackens. The appeal to the heart is not so much a gesture towards tenderness or the poetic but an approach to thought in the condition called delay that creates a stop in its movement, that brings disarray and a new form of relationality: ‘Severed from its impulse to self-reproduction, thought is momentarily related to the other’ ( Applebaum, 2001, p. 7). Thought as the ‘lurching gait of projection, the reaching back and throwing ahead’, and thought’s essential preoccupation with its own reproduction, is interrupted. Delay of the heart operates as a suspension of thought’s movement in order to bring on a new form of thinking.

Appelbaum’s appeal to the somatic, to the asynchronous force of something that offers a ‘sidewise’ approach to thinking that releases the habit of thought from its self-perpetuation, echoes a host of other philosophical perspectives – feminist, black feminist and Afro-pessimist perspectives in particular – that speak to the impossibilities yet necessities of remaining and dwelling in delay, not only as an ontology but as a politics and an ethics. Christina Sharpe, for instance, names this as ‘wake work’ ( Sharpe, 2016). ‘Wake work’ is the work it takes to go on living in the wake of the violence of slavery that cannot be overcome, where both mourning and melancholia are suspended, producing a time that must nevertheless be endured at a somatic and affective level in order that care as a form of thinking can emerge ^6^. The delay of the heart interrupts the violence of synchronous thought that seeks endlessly to reproduce itself, while refusing to ‘know’ about that violence. Such synchronous thought is violent to the degree that it denies the existence of what is outside itself and its own movements; it fails in its encounter with an other out of which something new, a new thought, could emerge. We could say that in this sense, delay – the suspension of time but also the suspension of the self-reproduction of thinking, of more of the same – holds open the possibilities for care for the future at the point that it can know about violence.

## Containment

If we can conceptualise delay as a form of care – one that suspends the impulse within thinking to reproduce itself in its failure to know about violence - can we turn this back to think about the question of containment, care and time? As we have seen, the UK Government’s initial ‘containment’ phase of its response to the outbreak of COVID-19 was over by the 12 ^th^ March. But the question of containment has not gone away, although it is now an issue focused more on the psychosocial than the microbiological. In conditions of forced isolation and social distancing, questions of how to contain anxiety and fear, of how to manage in the delay while knowing about violence, and of how to endure ourselves and others during this time of elongated waiting, have become pressing ^7^. A recent Review in *The Lancet* of studies of the psychological impact of quarantine concluded that where people are suddenly and forcibly rendered passive in relation to their circumstances, there is high prevalence of symptoms of psychological distress and disorder: emotional disturbance, depression, low mood, insomnia, post-traumatic stress symptoms, anger, emotional exhaustion and irritability ( Brooks *et al*., 2020). Qualitative studies of the SARS outbreak identified a range of other psychological responses, including confusion, fear, grief and numbness. *The Lancet* Review concludes that ‘the psychological impact of quarantine is wide-ranging, substantial, and can be long lasting’ ( Brooks *et al.*, 2020, p. 8), although it can be mitigated if people are kept informed about decisions taken and can understand and align their actions with them ^8^. Waiting in conditions of uncertainty becomes particularly disturbing or traumatic when our usual strategies for dealing with anxiety are removed and when uncertainty becomes overwhelming, as in situations where sources of income disappear overnight, when ‘safety nets’ seem unresponsive and require waiting far too long, and particularly when the ‘brick mother’ that is appealed to in the phrase ‘our NHS’ – an institution that can provide safety, care and a containment capable of holding us together when we are at our most vulnerable – is itself perceived to be under immediate existential threat ^9^.

There is a history we can draw on here that ties together a socio-historical literature on waiting during times of war in the twentieth century and the emergence of the concept of containment in psychoanalytic thinking in the British School of ‘object relations’ psychoanalysis. This latter tradition can be understood as a part of a wider attempt to use the relatively new discipline of psychoanalysis to understand and perhaps even mitigate the devastating violence of the two global, industrialised conflicts of the twentieth century ( Pick, 2014). In this psychoanalytic literature, distress, for example, is not simply imagined as the easily comprehensible result of experiences of anxious waiting under conditions of threat; rather, the difficulties of waiting become entangled with an understanding of psychological experiences in general and the management of violent and destructive instincts and drives. Suggestively, and as we will elaborate below, in this psychoanalytic literature the term ‘containment’ is used to represent what happens when unbearable and existentially threatening states of mind are understood rather than enacted. ‘Containment’, in this context, is also the prerequisite for the possibility of thinking that could allow itself to know about violence.

In 1940, during the waiting time of World War II known in the UK as the ‘Phoney War’ (when there were no major military land operations on the Western Front and no civilian experiences of aerial bombardment), the British psychiatrist and later psychoanalyst Wilfred Bion wrote a paper concerned with the inevitability of a devastating air-attack on London. There, he addressed the likelihood of civilian panic and the potential for an ‘epidemic of shell-shock’ comparable to what he had observed and experienced first-hand in World War I. Responding implicitly to Stanley Baldwin’s, 1932 statement that the experience of ‘total war’, in which military and civilian populations face devastating attacks from the air, was now inevitable – ‘the bomber will always get through’ – Bion wrote about the possibility of providing ‘psychological A.R.P.’ [air-raid precautions] ( Bion, 1940, p. 195). With the explicit aim of taking care of the civilian population newly exposed to military conflict, Bion suggested that people must not be left to languish in a kind of waiting time in which anxiety could take hold. Instead, as soon as an air-raid siren goes off, ‘[t]he alarm […] must be a call to action, and there must be an action to which every man and woman is called’ ( Bion, 1940, p. 189). In particular, Bion drew attention to the fact that isolation itself ‘can help produce that loss of social sense that is one of the characteristics of panic fear’ ( Bion, 1940, p. 185). Isolated and isolating waiting, which can lead to mental distress or what the later Bion described in 1962 as ‘a nameless dread’ ( Bion, 1984b, p. 116), must be replaced with communal, careful effort directed towards need in the present and the idea of a survivable future.

It is important both to note and trouble the ways that the current coronavirus crisis has been framed in the UK by looking back to World War II. In the second of his daily briefings on 16 March to the nation, Boris Johnson spoke of the need to take ‘steps that are unprecedented since World War II’ and of acting ‘like any wartime government’ ( Johnson, 2020a), reaching for a wartime imaginary in calls for national unity and resolve. For the British civilian population in general did not collapse in the face of aerial bombardment in the way many feared it might, although the idea that people did not experience psychological distress and lasting trauma from the Blitz was, first, useful propaganda (see *London Can Take It!*, 1940), and later a significant part of the mythology mobilised to shape ideas of postwar British exceptionalism. The establishment of the NHS in 1948 was also a direct response to the Beveridge report of 1942 that sought to produce a Welfare State capable of supporting reconstruction and aimed at rewarding national efforts and wartime sacrifice. The injunction to ‘save the NHS’, displayed prominently on the podium during the UK Government’s daily briefings, thus also makes a significant link back to that conflict and the postwar settlement.

Of course, the archive tells a more complex story of the reality of the waiting during World War II than a straightforward narrative of resolve and ‘pulling together’. Although admissions to psychiatric hospitals declined in 1940 in comparison to 1939 ( Jones, 2012, p.31), the detailed report of the psychological effects of bombing in the city of Hull ( Burney, 2012), for instance, demonstrated that experiences of fear and anxiety produced considerable and lasting trauma, if not total civilian collapse. Many people did make good use of the call to communal action, however: some by taking on roles on the Home Front explicitly associated with the war effort; others working at living on and getting on through domestic practices in which a relationship to an imaginable near future was maintained. This is matched in the present moment by the speedy emergence of community care networks, the revival of mutual aid groups and significant levels of volunteering to support NHS provision. As we write, the effects of a large-scale loss of life and its inevitable griefs and traumas, felt at both an individual and collective level, are breaking through the period of anxious waiting. Nevertheless, the traumatic effects of waiting and enduring through the lockdown persist alongside the imminence of existential threat. The desire both to ‘look after’ and be ‘looked after’ sits in a paradoxical relationship to modes where waiting – which might be care, but also might be violence and neglect – seems like the only thing to be ‘done’.

Between 1946 and 1952, Bion undertook an analysis with Melanie Klein, who had moved from Berlin to London in 1925. Bion went on to work closely with Klein’s idea of ‘projective identification’, which can be described as the way we may initially defend ourselves from impossibly difficult emotional experiences by temporarily splitting off undesired and sometimes valued parts of the personality and putting them into another person. For Klein and Bion, projective identification represented the lifelong repetition of experiences of early life in which the baby’s need, hate, love and its fear of death, were projected into a primary care-giver who would hopefully be ‘capable of reception of the infant’s projective identifications whether they are felt by the infant to be good or bad’ ( Bion, 1984b, p. 36). In receiving them in this way, the carer ‘contained’ and processed these elements – in Bion’s terms they ‘digested’ them – in a way that enabled the infant to feel it was ‘receiving its frightened personality back again but in a form it can tolerate’ ( Bion, 1984a, p. 115). For Bion, then, a crucial part of early development was the child's experience of care-givers who could be relied on to act as containers for their projective identifications and offer them back in forms that could be experienced as nourishing rather than destructive or contaminating.

Bion went on to represent projective identifications as particular kinds of thoughts that are full of feeling; indeed, he suggested in a 1962 essay that thinking evolves as a capacity for containing, absorbing and processing ‘thoughts’ otherwise experienced as intolerable. Bion believed, however, that such thoughts were vital communications that needed to be understood, and ‘containment’ became his term for the capacity of one individual (or a group or even an institution) to hear, absorb and work to understand the projections of another person as a meaningful communication. The task became to understand and convey these split off and projected thoughts back in a modified form that could, over time, be tolerated. For Bion, the aim of psychoanalysis was thus for analyst and analysand to suspend the unreflexive action that would risk getting rid of ‘thoughts’ experienced as contaminating or lacerating to the self and instead to hold, absorb and digest them over time and within psychical understanding. Containment became the process through which the analyst processed and gave back the feelings within thoughts as material with which one might think.

In his 1940 Penguin Special, *The Psychology of Fear and Courage*, the psychoanalyst Edward Glover described how humans can be like bombs: ‘people are charged with high explosives, in other words with very powerful, and sometimes uncontrollable, emotions’ and they ‘split where the cover is thinnest, that is to say, where our defences are weakest’ ( Glover, 1940, p. 27). Such a metaphor was timely for a book published as the ‘Battle of Britain’ was raging, but, even decades after both wars, Bion continued to describe ‘thoughts’ via an imaginary of bombs and missiles. For him, the only way to transform thoughts experienced as aerial bombardment was to suspend the mobilisation that sought to rid the psyche of them, what he called ‘evasion by evacuation’ ( Bion, 1984a, p. 117). Instead, he said that analyst and patient must learn together how to wait and to think, using time itself as a container. For if thoughts are ‘evacuated at high speed as missiles’ ( Bion, 1984a, p. 113), genuine thinking becomes a space of containment that allows the violence of the world to be taken into the self and digested over and through time, rather than unthinkingly expelled as invasive or intolerable. Such thinking, imagined according to the processes of a body able to digest rather than be torn open by explosive, incendiary ‘thoughts’, produces a space and time where violence might be suspended, delayed and therefore thought about, rather than simply enacted. Although there were practical benefits in encouraging communal, collective action to contain the anxiety of waiting time in wartime, for the later Bion it was waiting itself and thinking with others that came to be a ‘shelter’, a container, for an experience of time that enabled the possibility of an authentic ‘psychological A.R.P.’.

These ideas of containment as a capacity for ‘thinking’ brought on to deal with ‘thoughts’ experienced as violent attacks on the mind were born from particular scenes of anxious threat during the twentieth century, but continue to have significance for our current times, we believe. As we have noted, following Maria Puig de la Bellacasa, care time works to make time of a very particular kind, suspending the future and distending the present by ‘thickening it with myriad multilateral demands’ ( Puig de la Bellacasa, 2017, p. 207). As she puts it: ‘feelings of emergency and fear, as well as temporal projections, need often to be set aside in order to focus and get on with the tasks necessary to everyday caring maintenance’ ( Puig de la Bellacasa, 2017, p. 207). Feelings of emergency that can produce a panicked sense that any action is better than waiting, alongside more amorphous fears of what the future may or may not bring, both need to be wound back while focusing in the present on the needs of others if care, in the sense described above, is to be provided. Such care thickens the time of the present; nevertheless, it also retains a weakened commitment to the future – an ‘after’ into which selves and others are imagined as enduring. When linked to time, ‘after’ refers to a later, subsequent moment; but ‘after’, in many of its oldest usages, also means ‘behind’. ‘Looking after’ might be understood as a process of putting the object of one’s care ahead of one’s own position at the very moment one is positioned ahead. We might say it entails the delay of the heart. To ‘look after’ thus suggests the capacity to hold oneself back, to get behind those being cared-for, so that their needs can be responded to and they become the future towards one which is inclined. This is not any grand narrative of the future, but a rhythmic inclination that consists of persistent and persisting attention: a form of thinking that produces time that finds its place in the inter-generation, understood broadly, between self and others, as self finds a future in its relationship with another into which it might lean.

Significantly, the most recent NICE guidelines on the treatment of Post-traumatic Stress Disorder, including in the wake of major disasters, have reinforced the 2003 recommendations of the value of psychological containment and delay – of ‘watchful waiting’ ( NICE, 2003). ‘Watchful waiting’, in this context, steps back to enable immediate needs of shelter, food and clothing to be attended to. It does not offer complex psychological interventions too quickly; rather, it encourages the use of existing social and familial care networks for support, offering sufficient psycho-education and sense of presence through repetition to enable people to access specialised services if and when they are required ( NICE, 2003, p. 18). The evidence underpins the value of a strategic and thoughtful delay in action that requires services to contain their own anxiety and sense of emergency sufficiently that ‘thoughts’ might not overwhelm their ‘thinking’. Psychological therapies in this context might ‘look after’ us by both putting us ahead, while also waiting for us in our time of need. Such repetitions of care-ful attention and thinking, offered both as ‘watchful waiting’ and timely action, represent a commitment to a temporality of ‘looking after’; they affirm a belief in someone or something enduring through the bombardment of anxious ‘thoughts’ to produce a feeling of time that can be held on to long enough that it might be used.

## Mitigation – on not being able to touch

The final part of the UK government’s tripartite strategy is ‘mitigation’. It is triggered once a disease is widespread and it is no longer possible to either contain it or to slow its spread. Mitigation signals the belated shift to saving as many lives as possible and is the time of the most extreme measures coming into force: the use of the army on the streets to maintain public order; the closure of Parliament; the extreme enforcement of lockdown through centralised surveillance; and the rationing of care. Mitigation is an acknowledgement that containment and delay are no longer efficacious. If to mitigate is to attempt to make something already bad less severe, serious or painful, to lessen the gravity of an offence or mistake ( *OED*), then while it admits a tendency to enact violence at the very point that care is needed, it also contains a shadow of acknowledgment that a mistake and an offence against care has indeed occurred. Mitigation, as imagined in this strategy, runs the risk of inflicting ‘horrorism’ by rendering a vulnerable person helpless ( Cavarero, 2009, p. 50) at the very moment that help or care is required. But mitigation following an acknowledgment that an offence has taken place could also mean taking more care rather than less. In terms of COVID-19, mitigation might name a process of circling back in order to understand why some have been rendered more vulnerable than others, and to attend to that experience in the present with the aim of lessening suffering. Mitigating the suffering of COVID-19 could also mean committing to understanding vulnerability as always already relational, a feature of ‘our shared or interdependent lives’ ( Butler, 2019, p. 45), in order to put in place collective measures that would open up different futures in a pandemic that has not yet run its course. A future born from a present committed to mitigation could be one in which lives lost would be ‘grievable’, vulnerability would be placed ahead, and acting in the name of containment and delay would open up time for care, mitigating the slippage of vulnerability into helplessness.

In a healthcare context we can talk of mitigating pain, where pain is not just an offence to the body but includes the pain of psychological, social and spiritual suffering – what Cicely Saunders, the founder of the Hospice movement, named ‘total pain’. One of the core principles of palliative care is the refusal to separate bodily pain from its other social and cultural determinants when offering holistic mitigation of suffering at the end of life. As Yasmin Gunaratnam notes in *Death and the Migrant* (2013), although ‘pain needs a body’, relying on flesh ‘to register and receive it’ and ‘allow it passage’ (p. 133), it arises from multiple, often unacknowledged sources. In tracing the stories of ageing and dying in the health service for those who have migrated to the UK since World War II, including the many migrants who have cared for others within the health service (a disproportionate number of whom are now dying of COVID-19), she brings together Pierre Bourdieu’s notion of social suffering ( Bourdieu, *et al*., 1999) with Saunders’ account of ‘total pain’ ( Saunders, 1967). In doing so she works to recognize how pain is accrued and suffered over a lifetime ( Gunaratnam, 2013, p. 137). Following this, we would suggest that mitigation, as a form of palliative care, needs to attend carefully to the total pain of COVID-19, and the conditions of radical uncertainty it produces, in ways that can respond to the multiple. This would acknowledge the suffering of individuals in the present, but would not erase the cumulative effects of ongoing racism and social inequality, the brutalities of neoliberalism that have damaged working conditions in the NHS and its capacity to care ^10^, and the ongoing human-induced loss of habitats for non-human animals that have increased the likelihood of zoonotic disease transfer. All of these determinants, and more, find their ‘body’ in the person dying of COVID-19.

One of the threads that runs through *Death and the Migrant* is the social and political life of touch. Gunaratnam describes how in so many instances, care at the end of life entails profound experiences of touch – of washing and being washed, of being held, handled and caressed, of using the hands to express total pain by ‘praying’ through handling a rosary or ‘mala’. These experiences of touch counter the numerous forms of intrusive touch that also accompany illness and the end of life: being prodded and poked and instances of unwanted touch – experiences that are always already gendered and raced. Touch may be delivered violently; it might also be withheld as care fails. However, Gunaratnam draws our attention to the value of touch in cross-cultural palliative care as something that materialises a particular kind of ‘looking after’ when language and established procedures cannot necessarily make sense of what is needed. She writes:

Radical doubt and uncertainty are not unique to cross-cultural palliative care. They can surface in situations where routines of care become ineffective, where trust and communication breaks down and professionals have to work out and improvise not just what to do, but also what kind of care they want to create and be part of. ( Gunaratnam, 2013, p. 101)

Touch, offered in the space and time of radical uncertainty, speaks of the potential for new possibilities of mitigation and containment to be found; it also speaks of the permanent possibility that care might fail.

Perhaps one of the most difficult stories to emerge in the UK press to date has been the death of Ismail Mohamed Abdulwahab, who on the 1 ^st^ April 2020 was the youngest person in the UK to die of COVID-19. What made his death particularly painful to know about was not just how young he was, and the inexplicability of why a young boy who appeared to have no underlying health conditions should die of COVID-19, but that he died alone. Because of the risk to his family’s health, it was advised that he could not be touched, held and comforted by those who loved him as he died. For patients in an induced coma on a ventilator in intensive care, this form of touch was initially prohibited, although on 15 ^th^ April the UK Health Secretary described being ‘moved’ by stories of people dying alone and introduced new guidelines ( Hancock, 2020). These guidelines permitted physical presence that would give ‘people the chance to say goodbye’, while attempting to mitigate the risk of infection. But care in conditions of radical uncertainty has also been offered in other ways – in the form of FaceTime or Zoom contact, for instance, which sometimes offers containment and mitigation of pain and sometimes fails. In the absence of routines of care that would usually involve physical proximity, we are being pushed to improvise and to decide what kind of care we want to create and be part of.

The psychoanalytic literature makes much of the importance of physical proximity and analyst and patient meeting and waiting together regularly. It is the regular repetition of the act of understanding and containment that produces the conditions in which thinking can take place and time can potentially be used rather than got rid of in unthinking action. Instead of waiting *for* something specific to happen, the emphasis is on waiting *with* and responding, in the present, and in the time of thinking, to the anxious bombardment of thoughts whose qualities can become knowable, in all their difficulty and violence. But how can we wait *with* one another under the conditions of a profoundly unknown and unknowable future; how can we wait together when physical proximity is the thing that must be avoided? It is perhaps worth remembering that, almost from the very beginning, psychoanalysis has taken place under the agreement that there should be no physical contact between analyst and patient ^11^. This idea of holding in mind, emphasised by psychoanalyst D. W. Winnicott (1960), alongside Bion’s notion of psychological containment, indeed emerged in the name of an offer of contact through understanding rather than via material touch. But under material conditions of lockdown, or those that require a two-metre space between people, the idea of ‘holding’ in space might be less psychologically useful than the idea that there may be ways in which unbearable fear and anxiety can be contained within *time*. In a psychic imaginary now dominated by fears of contagion, of being invaded by ‘thoughts’ and anxieties that are as ‘viral’ as COVID-19 in their capacity to spread and to seep through domestic and bodily borders, a form of holding might still be able to occur through a sharing of verbal and embodied communication in time – a being *with* that enables containment in and of time.

How, then, might we think of using contact in time, a waiting *with* in time, as a way of containing the waiting time that COVID-19 has demanded of entire populations? It is clear that virtual environments are already enabling some people to remain in contact in time. From psychoanalysis to religious gatherings to birthday parties – communication technologies have been making more bearable the requirements of isolation and social distancing that might otherwise be experienced as intolerable, even as the withdrawal of touch for some communities produces losses that simply cannot be mitigated (see Anonymous, 2020b). For those able to use these technologies, the greater challenge might be remaining in contact *with time itself*, particularly with the time of waiting and delay. Waiting can be experienced as an intolerable impingement on freedom; it can also be easy for distraction to dominate when much of life starts to be lived online. Feelings of lack of agency can produce obsessive rituals of checking information that work as attempts to reinstate a feeling of time’s forward movement, but only fill the present by filling in for time’s ever-weakening dynamism (see Salisbury & Baraitser, 2020). Isolation and social distancing are also palpably intensifying for some the demands of those sectors of the economy that were already or have been able to move swiftly online. Following the clear trends of neoliberal labour practices in which responsivity, availability and forms of affective labour have replaced clocking on and off, the sensation of capital occupying all areas of human life and of endless busyness has not left many of those whose work is deemed to be able to continue online. At the same time, those whose work outside of the home is deemed ‘essential’, alongside many populations who live without the privilege of conditions that would enable social distancing or self-isolation to take place, endure the exhausting practices and anxieties associated with attempting to mitigate the essential vulnerability produced by coming into physical contact with others. While many people remain contained, more or less tolerably in more or less impermeable spaces, others face the discomfort, sometimes the agony, of containing their anxieties and using practices of decontamination and the physical barriers of Personal Protective Equipment to mitigate the fact that bodies are not impermeable and that contact with others is essential for material care.

It is understandable that the temporality of the urgent might be prioritised in the current circumstances. There are immediate needs and demands that need to be cared for. But it is also clear that the call to action that Bion suggested in 1940 was implicitly a call to thoughtful action rather something that might be used as an evasion of thinking. For Bion, thoughtful action required what he later called ‘patience’ – the possibility of containing the anxiety of uncertainty and using it instead as the ground for the possibility of thinking. As he went on to suggest, there is always pressure to ward off the uncertainty of not knowing by leaning on prior knowledge despite those circumstances no longer obtaining, or adopting a new certainty too quickly while excluding other elements that might bring a new pattern of meaning into view ( Bion, 1970, p. 124). As Steve Hinchliffe (2020) has argued, the understandable tendency in the present COVID-19 crisis to lean on particular kinds of epidemiological models of evidence, partially because they have the virtue of imagining futures that seem potentially knowable and can be relatively simply communicated, risks filtering out other forms of experiential evidence that might be important in shaping an effective response to an ongoing and evolving situation. The capacity to keep thinking under conditions of radical uncertainty, to be open to the unknown and to the complexity of the present moment, to be open to the possibility of others futures where priorities for care might be fundamentally rethought, can seem almost impossible when the pressure is on to act, to mitigate in conditions of urgency. Nevertheless, for Bion an openness to what is unknown enables a relationship between, rather than a confusion of, internal and external reality, and the formation of an alliance for thinking made in contact others that could suspend action until it is thinking’s precipitate, rather than its substitute.

## Conclusions

The UK’s plan to follow a strategy of ‘containment, delay and mitigation’ implies a linear, progressive temporality, even though it has been clear, almost from the beginning, that the idea of moving from one phase on to another does not map easily on to the complex reality of a pandemic. At an explicitly political level, as the experiences of South Korea and Germany are suggesting, delay and mitigation should not be thought of as simply superseding strategies of containment – testing, quarantine and contact tracing – even after it is clear that the virus is spreading in the community and even though such containment is resource heavy. Maybe it is obvious that containment of the virus can never be separated from the need to delay and to mitigate. But perhaps it needs to be reaffirmed at this point that any future mitigation must not throw aside all attempts to stay with practices of care that seek to contain and delay cases of COVID-19, if it is not to inflict ‘horrorism’ and abandonment at the moment when care is still needed.

We have argued here that by thinking the terms containment, delay and mitigation through in psychosocial terms and within a more enfolded and recursive temporality, we might be able to keep more in touch with and learn something from the failures that are always a possibility within any caring encounter. To be explicit, this requires thinking the temporality of the response to COVID-19 in a more care-ful fashion, as a time that would enable the figures of containment, delay and mitigation to hover and adjust themselves in relation to one another. Our point is that this more recursive temporality of repeating and returning is likely to be able to know more about ongoing violence as it holds back from narratives of battles to be won. Such a temporality might, in turn, allow us to know more about the ever-present possibility of failures of care that get written out of discourses of healthcare heroism – to know how such failures occur, what they might communicate and something about how such failures could be contained, delayed or mitigated. Writing between March and May 2020, we have circled back to a point in twentieth-century history that was also concerned with an existential threat requiring a response from a whole population, but we have done this not in the name of invoking a fantasised narrative of ‘Blitz spirit’. Instead, we have suggested that the British psychoanalytic tradition born of that moment insisted that one must keep thinking while ‘under fire’ and that there are possibilities of containing unbearable anxiety and the capacity for violence in the intersubjective space and time between people. This commitment to thinking in and with the process of delay and containment might yet be drawn upon as we inhabit this time of waiting – waiting that is the management and mitigation of a future threat, but also a time of care in and for the present.

## Data availability

All data underlying the results are available as part of the article and no additional source data are required.

## Notes

^1^See Flexer, 2020.

^2^See Bivins *et al.*, 2018. See, also, Moore, 2020.

^3^See https://assets.publishing.service.gov.uk/government/uploads/system/uploads/attachment_data/file/874290/05-potential-impact-of-behavioural-social-interventions-on-an-epidemic-of-covid-19-in-uk-1.pdf

^4^See, for instance, Anthony Costello’s frequent contributions to The Guardian.

^5^See Care Collective, 2020.

^6^See also the work of Fred Moton on ‘fugitivity’ and living ‘in the break’ ( Moton, 2003); Best and Hartman on Fugitive Justice ( Best & Hartman, 2005); and Frank, B. Wilderson III on Afropessimism ( Wilderson, 2020).

^7^A recent Ipsos MORI poll has suggested that people are more concerned by the effects of social and psychological response to the pandemic than the physical illness COVID-19 ( Holmes *et al*., 2020, pp. 1–2).

^8^This matches Healthwatch’s, 2019 submission to NHS England on people’s views on A&E waiting times. The report concluded that those who are triaged on arrival, have the next steps explained to them and are kept informed of changes due to other circumstances, are more positive about their experience, even if they end up waiting a long time.

^9^The term ‘brick mother’ was used by Henri Rey, who had a strong influence on trainee psychiatrists in the Maudsley Hospital in South London in the post-World War II period. For him, the term evoked an idea of safety, continuity, stability and asylum – in its true sense – for patients who were frightened of breaking down. For further on the sense of the NHS under threat, see Moore, 2020.

^10^See, also, Davies, 2020.

^11^Freud describes using techniques of hypnosis and touch in *Studies in Hysteria*, but stated in 1893 that the ‘talking cure’ should be undertaken without physical examination or the laying on of hands (see Freud, 1893).
