# Supplementary material for: ‘Containment, delay, mitigation’: waiting and care in the time of a pandemic
Source: Wellcome Open Res. 2020 Sep 10;5:129. Originally published 2020 Jun 10. [Version 2] doi: 10.12688/wellcomeopenres.15970.2 (PMC7361498; doi:10.12688/wellcomeopenres.15970.2)
Supplement: Peer review history

# Peer review: Reviewer response for version 1

**DOI:** 10.21956/wellcomeopenres.17514.r39136

**Published:** 2020-07-14

**Authors:** Melissa Jacques

**Affiliations:** 1 University of British Columbia, Kelowna, Canada

Lisa Baraitser and Laura Salisbury take up the three terms used in the UK government’s political strategy for dealing with the Covid-19 pandemic - containment, delay, mitigation - and subject these terms to a psychosocial reading. What this translation makes possible is the production of the time and space necessary for thinking through the relation between care and violence, and between thought and action, in the midst of the current crisis. By refusing a teleology that frames the disease in terms of a forward-moving momentum leaving little time for reflection, they argue for a recursive response, one that more accurately mirrors the “complex trajectories” of the disease itself. Through a series of iterative meditations, they invite their readers to pay attention to the ways that “care” might result in failure, for example, when “delay” becomes abandonment and those most in need of care are subjected to the violence of care’s failure.

 Making their arguments in concert with a range of voices - from Jacques Derrida and Judith Butler to British psychoanalysis and Adriana Cavarero’s thinking through of “horrorism” - Baraitser and Salisbury explore the generative possibilities of delay and call for a rupture within self-reproducing thought that makes room for what David Applebaum calls the “delay of the heart”. Within this rupture, they argue, we might be able to live through the crisis of the pandemic while at the same time confronting the violence of governmental responses that have resulted in a failure of care. It is only through this “care-ful” attention to systemic violence that we might move forward into a survivable future. They conclude with a section on mitigation that revolves around the experience of touch. Framing touch as having the potential to do violence as well as bring comfort, they nevertheless suggest that there is hope in proximity as it holds the promise of alliance, a bridge between the internal and external, and a placing the self and other in more ethical relation.

 I am both moved and convinced by Baraitser and Salisbury’s intervention and have only one suggestion. In *Precarious Life* (2004), Judith Butler confronts the ethical failures of a regime that deems certain lives grievable and others dispensable. She is also critical of a government that refuses to give grief its necessary time in favour of pursuing unreflective violent action. It seems that we are being returned to this earlier moment and being given yet another opportunity to confront familiar forms of violence.

Is the work clearly and accurately presented and does it cite the current literature?

Yes

If applicable, is the statistical analysis and its interpretation appropriate?

Not applicable

Are all the source data underlying the results available to ensure full reproducibility?

No source data required

Is the study design appropriate and is the work technically sound?

Yes

Are the conclusions drawn adequately supported by the results?

Yes

Are sufficient details of methods and analysis provided to allow replication by others?

Yes

Reviewer Expertise:

Critical and cultural theory, narrative medicine, medical humanities.

I confirm that I have read this submission and believe that I have an appropriate level of expertise to confirm that it is of an acceptable scientific standard.

## References

[1] : Precarious Life:The Power of Mourning and Violence. *Verso*.2004;

# Peer review: Reviewer response for version 1

**DOI:** 10.21956/wellcomeopenres.17514.r39038

**Published:** 2020-07-02

**Authors:** Rebecca Coleman

**Affiliations:** 1 Sociology Department, Goldsmiths, University of London, London, UK

This is a thoughtful analysis of the UK government’s phased response to the COVID-19 pandemic, which elaborates the temporal dimensions of ‘containment: delay: mitigation’ through a focus on care. The paper draws attention to the, often overlooked, centrality of time to the pandemic, analysing closely the intricacies of the different phases and their implications for how care is (and isn’t) done, and for the possibilities of doing care otherwise. The argument is sophisticated and nuanced. It draws out the specificities of time and care to understanding COVID-19 and also, in my opinion, makes points that have wider resonance – for example, with respect to how time and care work more generally in the NHS, palliative care and in psychoanalytic encounters. Indeed, a real strength of the piece is in its contextualisation of the responses to the pandemic within longer histories of care in medicalised situations. This focus on care and time is original and makes important contributions to understandings of health and medicine. I appreciated the argument that time must be understood as recursive and non-linear, and how this was embedded in the structure of the discussion which begins with delay in order to understand containment and mitigation. The literature that is drawn on is appropriate to the authors’ concerns and is mobilised in the service of a novel argument (rather than relied upon to make an argument).

 I have very few comments or suggestions on the paper, as I found it compelling and persuasive. The argument is complex as is appropriate to the topic and aims of the piece. I wondered whether the authors might explicate a couple of points of discussion in order to provide a bit more detail for readers unfamiliar with some of the literature. For example, Cavarero’s argument about ‘horrorism’ seemed important to the argument; could the discussion of it be expanded? On this point, I also wondered whether it could be pulled through in the latter parts of the paper, especially as it is mentioned in the abstract. Similarly, regarding the definition of mitigation as an acknowledgment that care has not been sufficiently taken, is this of any relevance to the broader argument about touch, holding and waiting developed in this section? In other words, I wondered whether a bit more could be made of mitigation, not only as a phase in the response to COVID-19 but in what it refers to and signals. I think this point is latent in the argument and could perhaps be drawn out further.

 These comments are made in the context of what is an excellent article. I am looking forward to reading the paper again once it is finalised.

Is the work clearly and accurately presented and does it cite the current literature?

Yes

If applicable, is the statistical analysis and its interpretation appropriate?

Not applicable

Are all the source data underlying the results available to ensure full reproducibility?

No source data required

Is the study design appropriate and is the work technically sound?

Yes

Are the conclusions drawn adequately supported by the results?

Yes

Are sufficient details of methods and analysis provided to allow replication by others?

Yes

Reviewer Expertise:

Cultural theory, feminist theory.

I confirm that I have read this submission and believe that I have an appropriate level of expertise to confirm that it is of an acceptable scientific standard.
